# A retrospective study of 1064‐nm Q‐switched Nd:YAG laser therapy for acquired bilateral nevus of Ota‐like macules

**DOI:** 10.1111/srt.13298

**Published:** 2023-03-06

**Authors:** Xinjun Yang, Chen Bi, Tianyu E, Li Lin, Yongqian Cao

**Affiliations:** ^1^ Department of Plastic and Aesthetic Surgery Shandong Provincial Hospital Affiliated to Shandong First Medical University Jinan Shandong P. R. China; ^2^ Department of Plastic and Aesthetic Surgery Shandong Provincial Hospital, Cheeloo College of Medicine, Shandong University Jinan Shandong P. R. China

**Keywords:** 1064‐nm Q‐switched Nd:YAG laser, acquired bilateral nevus of Ota‐like macules, melasma, postinflammatory hyperpigmentation, related factors

## Abstract

**Background:**

The therapeutic efficacy of laser treatments for acquired bilateral nevus of Ota‐like macules (ABNOM) varies among studies, and few studies have evaluated the factors affecting therapeutic effects.

**Aims:**

To evaluate the efficacy and safety of 1064‐nm Q‐switched Nd:YAG laser (QSNYL) therapy for ABNOM and to identify the factors influencing the outcome.

**Methods:**

A total of 110 patients with ABNOM were retrospectively evaluated and received two‐to‐nine treatment sessions. The effects of different factors on the therapeutic effect were analyzed on the basis of the number of treatments, age at first treatment, skin type, lesion color, affected area, number of lesion sites, and presence of concomitant melasma.

**Results:**

The curative effect was positively correlated with the treatment time and negatively correlated with the increasing age at first treatment (*p* < 0.05). The curative effect was better in patients with skin type III than those with type IV ( *p* < 0.05) and in patients with a lesion area of less than 10 cm^2^ than those with a larger affected area (*p* < 0.05). Additionally, the treatment effect was poorer in patients with concomitant melasma (*p* < 0.05). The treatment effect was not significantly correlated with the lesion color or number of affected sites (*p* > 0.05). Eleven patients (10%) developed postinflammatory hyperpigmentation (PIH).

**Conclusions:**

Early and repeated QSNYL therapy achieved satisfactory results for ABNOM. The risk of PIH after laser treatment is highest among patients with older age, darker lesion color, and darker skin color. For patients with ABNOM with concurrent melasma, low‐energy laser therapy is recommended to reduce the risk of melasma aggravation.

## 1 INTRODUCTION

Acquired bilateral nevus of Ota‐like macules (ABNOM) was previously considered to be a variant of nevus of Ota.^1^ ABNOM often occurs in the zygomatic region and is also called nevus fusco‐caeruleus zygomaticus.^2^ As an acquired disease, ABNOM is more common in Asian women older than 20 years. Most cases are bilaterally symmetric and do not involve mucosa. Histopathological examination shows slender, pigment‐bearing cells dispersed in the middle and upper parts of the dermis, with some melanocytes distributed in the perivascular area.^1^, ^2^, ^3^

Various types of lasers have been used to treat ABNOM, including the Q‐switched ruby laser (QSRL),^4^ Q‐switched alexandrite laser (QSAL),^5^, ^6^, ^7^, ^8^ Q‐switched Nd:YAG laser (QSNYL),^9^, ^10^, ^11^, ^12^, ^13^ and picosecond alexandrite laser (PSAL).^14^ QSRL is an effective and less invasive tool for the eradication of NFZ. But it has the risk of long‐term hypopigmentation.^4^ After QSAL treatment, there is a high probability of erythema and hyperpigmentation or hypopigmentation.^5^ Yu et al. showed that PSAL seems to have better efficacy and safety than QSAL, but there are few studies on the treatment of ABNOM with PSAL, which needs further discussion.^14^ Epidermal pigmented lesions respond best to 532‐nm QSNYL with shorter wavelengths. ABNOM, as a dermal pigmented lesion, has not been reported to be treated with 532‐nm QSNYL alone. Compared with other lasers, the 1064‐nm QSNYL has a longer wavelength and selectively targets deeper tissues, and it has poor absorption in epidermal melanin, making it relatively safe for people with dark skin.^15^ Although several studies have evaluated the 1064‐nm QSNYL in the treatment of ABNOM, the treatment effect is inconsistent and the related factors affecting the efficacy are also controversial. To improve the efficacy and optimize the treatment strategy, we retrospectively analyzed the factors affecting the results of 1064‐nm QSNYL therapy for ABNOM.

## 2 MATERIALS AND METHODS

### 2.1 Patients

All patients with ABNOM who underwent at least two treatments with 1064‐nm QSNYL alone in the Department of Laser and Aesthetic Medicine of our hospital from March 2019 to June 2022 were retrospectively studied. For patients concomitant melasma, different diagnoses were made at the initial visit. Contraindications to treatment were as follows: (1) pregnancy or breastfeeding; (2) photosensitive diseases or serious systemic diseases; (3) recent use of photosensitive drugs or food or exposure to sunlight; (4) oral intake or external application of retinoids within the last 6 months; (5) application of freckle‐removing products, laser, freezing, or other freckle‐removing treatments in the last 6 months; (6) infection or active skin lesions at the treatment site; and (7) propensity to scarring. All patients initiated their first treatment in this study. Before the first treatment, patients were given verbal and written information about the treatment process, potential complications, and therapeutic alternatives. All patients or parents of the child participants provided written informed consent. Data were obtained regarding sex; age; Fitzpatrick skin types III–IV; lesion color and area and number of lesion locations; induction of new melasma lesions and worsening of existing melasma lesions; postinflammatory hyperpigmentation (PIH); and number of laser treatments and degree of improvement of lesions after treatment. This study was approved by the Ethics Committee in our institute according to the Declaration of Helsinki principles (SWYX: no. 2022‐171).

### 2.2 Treatments

Before treatment, each patient's face was cleaned and photographed with Visia. Compound lidocaine cream was applied to the lesions at least 40 min before treatment. After removal of the lidocaine cream, test spots were used to determine the threshold fluences on each patient and define treatment endpoints (appearance of slight whitening without bleeding and tissue splatter). Treatment was performed using the MedLite C6 QSNYL (Cynosure, USA) with a wavelength of 1064 nm, spot size of 3–6 mm, energy density of 2.8–8.0 J/cm^2^, and pulse frequency of 2.0–5.0 Hz. The treatment parameters were selected individually, and the interval between treatment sessions was 3–6 months. For the lesion concomitant melasma, the fluence is reduced to 2.8–4.0 J/cm^2^. Appropriate eye protection was worn, whereas the laser was in use. Ice packs were applied for 0.5 h after each laser treatment session to reduce thermal damage. A topical antibiotic ointment (Mupirocin) was applied twice a day for 3 days. Patients were instructed to practice strict sun protection.

### 2.3 Visual evaluation

The pre‐ and posttreatment Visia photographs were compared using visual evaluation. The quartile improvement scale was used to assess the pigmentation clearance in the treated areas at 6 months after the final treatment as poor (0%–25%, score 1), fair (26%–50%, score 2), good (51%–75%, score 3), or excellent (76%–100%, score 4) (Table 1). ABNOM lesions were assessed by two plastic surgeons who were not involved in the treatment. When the two assessors reported different improvement scores for a given lesion, the lower of the two scores was used. Any adverse effect such as erythema, pigmentary changes, or scarring was recorded.

**TABLE 1:** Quartile improvement scale after treatment.

Quartile improvement scale	Number of patients, *n* (%)
1 = poor (0%–25%)	15 (13.6)
2 = fair (26%–50%)	32 (29.1)
3 = good (51%–75%)	33 (30.0)
4 = excellent (76%–100%)	30 (27.3)

### 2.4 Statistical analysis

Statistical analysis was performed using SPSS version 26.0. The Mantel–Haenszel *χ* ^2^ test was used to determine whether there was a linear relationship between two factors. The predictors were lesion color, area, number of lesion sites, and combined melasma; the outcome measure was age. The *χ* ^2^ test was also used to identify the factors affecting PIH. The univariate analysis (*χ* ^2^ test) and multivariate logistic regression analysis were used to analyze the factors affecting the efficacy of 1064‐nm QSNYL therapy in the treatment of ABNOM. Variables with *p* < 0.10 in univariate analysis were included in the model for stepwise multivariate logistic regression analysis, and a two‐sided *p* value of <0.05 was considered statistically significant.

## 3 RESULTS

### 3.1 Patient characteristics

**FIGURE 1:**
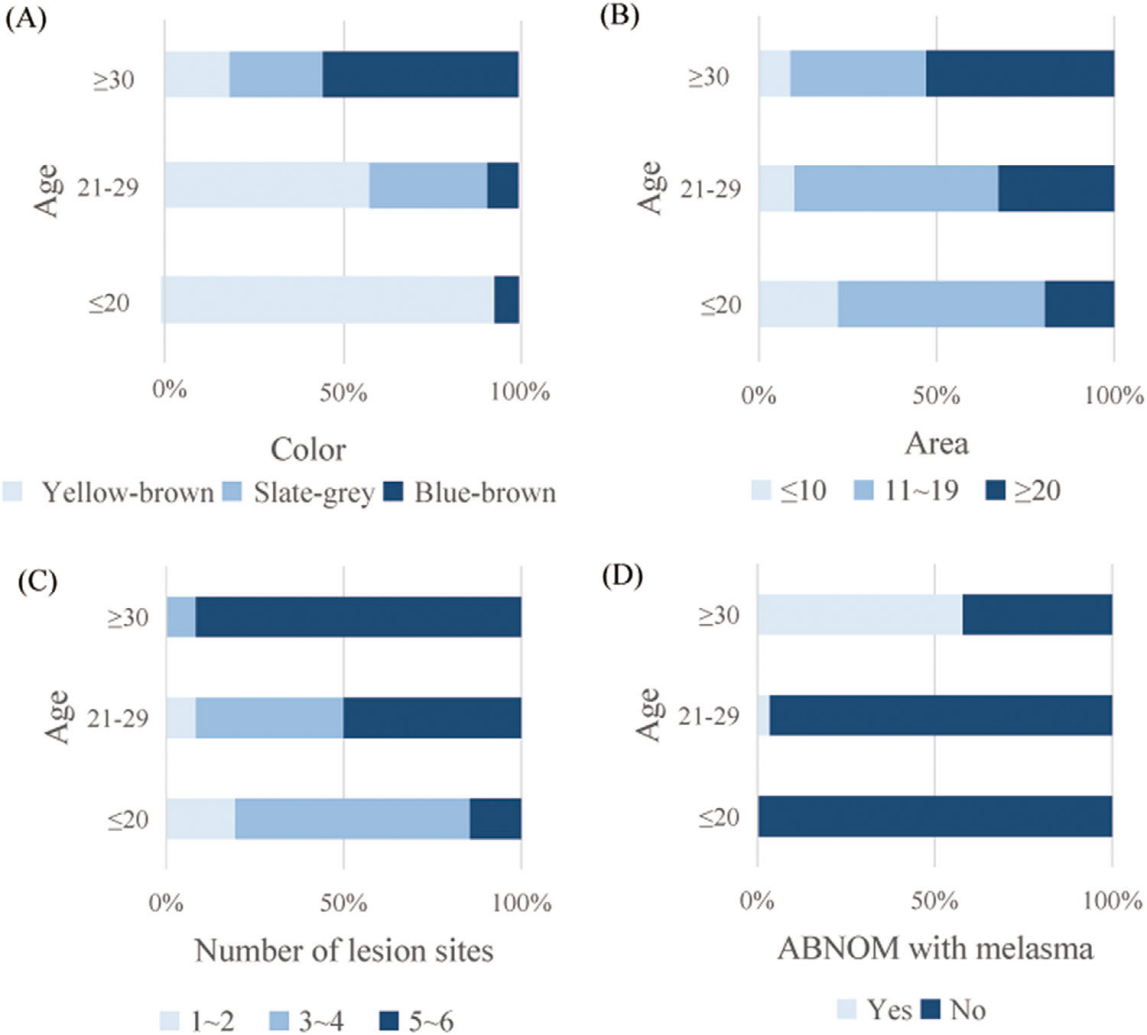
Assessment of the linear relationship between each factor and age using the Mantel–Haenszel *χ* ^2^ test. (A) Lesion color in various age groups. The lesion color increased with age (*χ* ^2^ = 32.411, *r* = 0.545, *p* < 0.001). (B) Lesion area in various age groups. The lesion area increased with age (*χ* ^2^ = 8.714, *r* = 0.283, *p* = 0.003). (C) Number of lesion sites in various age groups. The number of lesion sites increased with age (*χ* ^2^ = 26.381, *r* = 0.492, *p* < 0.001). (D) Prevalence of concomitant acquired bilateral nevus of Ota‐like macules (ABNOM) and melasma in various age groups. The probability of ABNOM combined with melasma increased with age (*χ* ^2^ = 22.496, *r* = 0.454, *p* < 0.001). *p* < 0.05 was considered statistically significant. ABNOM: acquired bilateral nevus of Ota‐like macules; *r*: Pearson correlation coefficient.

A total of 110 patients with ABNOM were included (108 females and 2 males). According to the characteristics of the onset age of ABNOM and melasma, the age is divided into 20 years or younger, 21–29 years, and 30 years or older. The patient characteristics are summarized in Table 2. The zygomatic region was affected in all 110 patients. Lesion color was positively correlated with age (*χ* ^2^ = 32.411, *p* < 0.001); as the patient age increased, the lesions gradually became gray (Figure 1A). There were also positive correlations between age and lesion area (*χ* ^2^ = 8.714, *p* < 0.05) and between age and number of lesion sites (*χ* ^2^ = 26.381, *p* < 0.001) (Figure 1B,C). Eighteen patients (16.4%) had both ABNOM and melasma, and the risk of melasma increased with age (*χ* ^2^ = 22.469, *p* < 0.001) (Figure 1D).

**TABLE 2:** Patient demographic data.

Study period	2019.3–2022.6
Population studied	Chinese
Age of first treatment range (mean ± SD), years	5–48 (27.59 ± 8.43)
≤20, *n* (%)	15 (13.6)
21–29, *n* (%)	57 (51.8)
≥30, *n* (%)	38 (34.5)
Gender, *n* (%)	
Male	2 (1.8)
Female	108 (98.2)
Fitzpatrick skin type, *n* (%)	
III	45 (40.9)
IV	65 (59.1)
Color of the lesions, *n* (%)	
Yellow–brown	54 (49.1)
Slate–grey	29 (26.4)
Blue–brown	27 (24.5)
Number of lesion sites, *n* (%)	
1–2	62 (56.4)
3–4	36 (32.7)
5–6	12 (10.9)
Area of lesions, range (median, IQR) (cm^2^)	3–28 (14, [10–20])
≤10, *n* (%)	36 (32.7)
11–19, *n* (%)	40 (36.4)
≥20, *n* (%)	34 (30.9)
ABNOM with melasma	
Yes	18 (16.4)
No	92 (84.6)
Total	110 (100.0)

Abbreviations: ABNOM, acquired bilateral nevus of Ota‐like macules; IQR, interquartile range; SD, standard deviation.

### 3.2 Clinical efficacy

**FIGURE 2:**
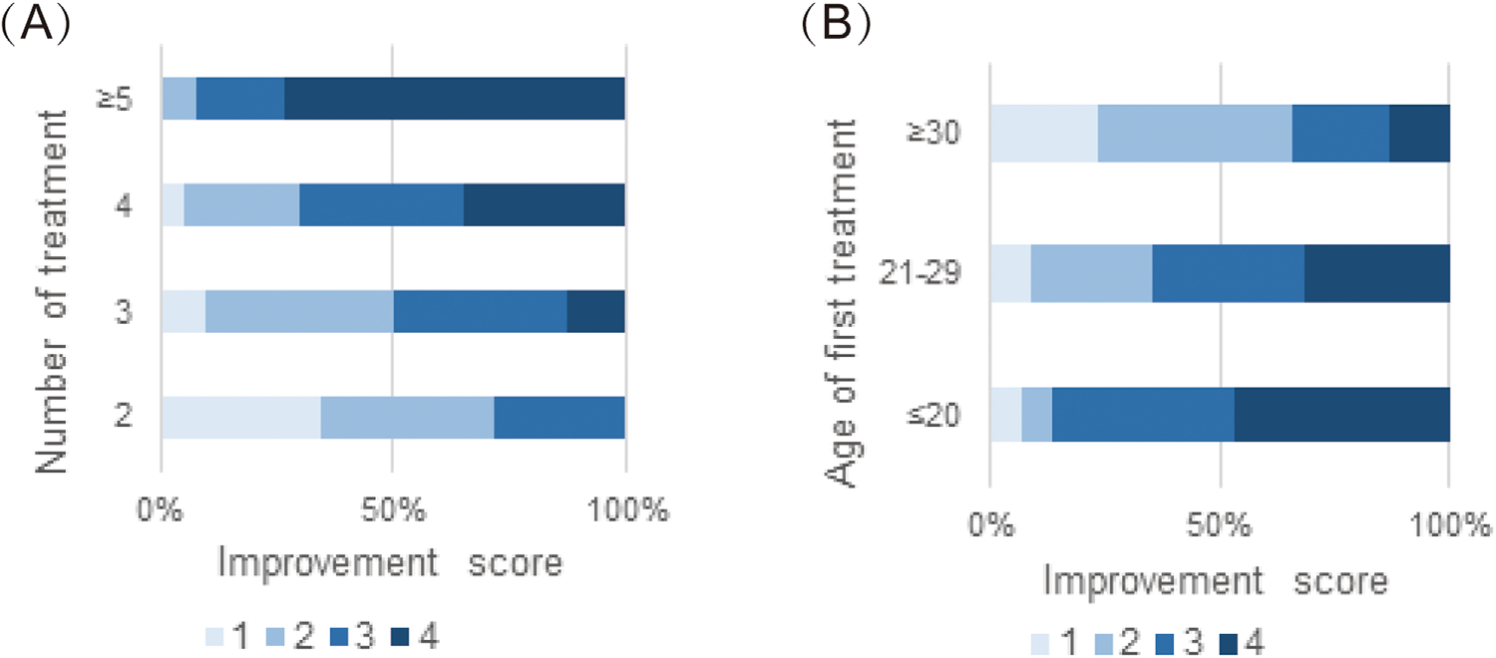
Assessment of the linear relationship between each factor and the improvement score using the Mantel–Haenszel *χ* ^2^ test. (A) Lesion improvement scores in the groups with various numbers of treatment sessions. The improvement score increased as the number of treatment sessions increased (*χ* ^2^ = 43.365, *r* = 0.631, *p* < 0.001). (B) Lesion improvement scores in various age groups. The lesion improvement score decreased with age (*χ* ^2^ = 13.893, *r* = −0.357, *p* < 0.001). *p* < 0.05 was considered statistically significant. *r*: Pearson correlation coefficient.

**FIGURE 3:**
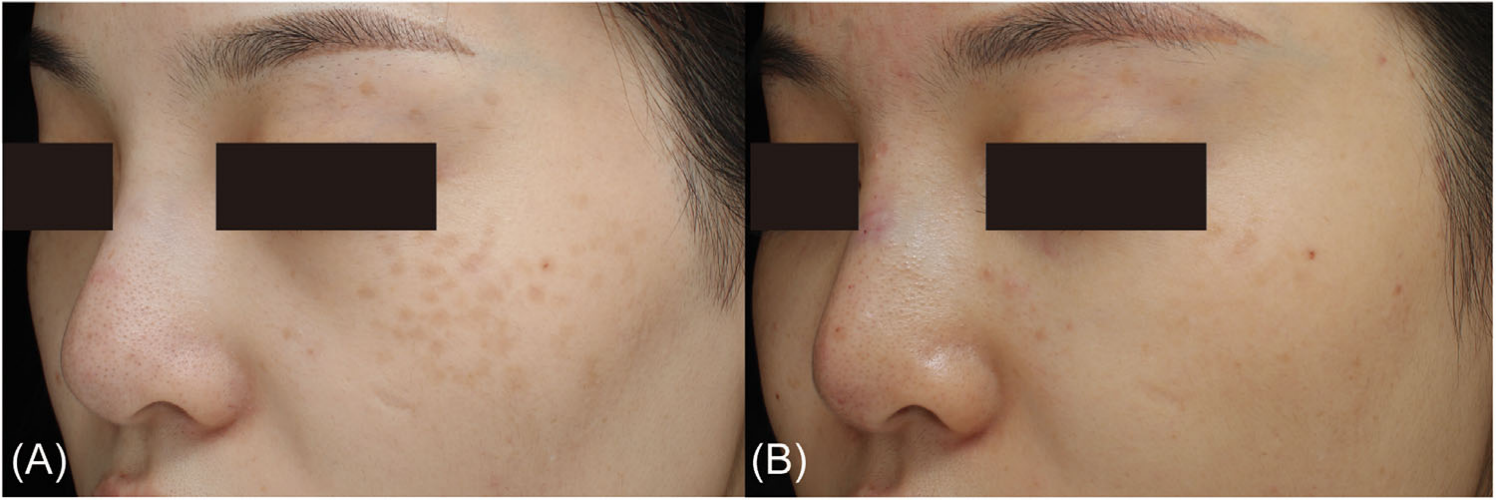
Photographs of a woman in her 20s with only acquired bilateral nevus of Ota‐like macules. (A) Before treatment. (B) Disappearance of all lesions after the sixth treatment session.

**FIGURE 4:**
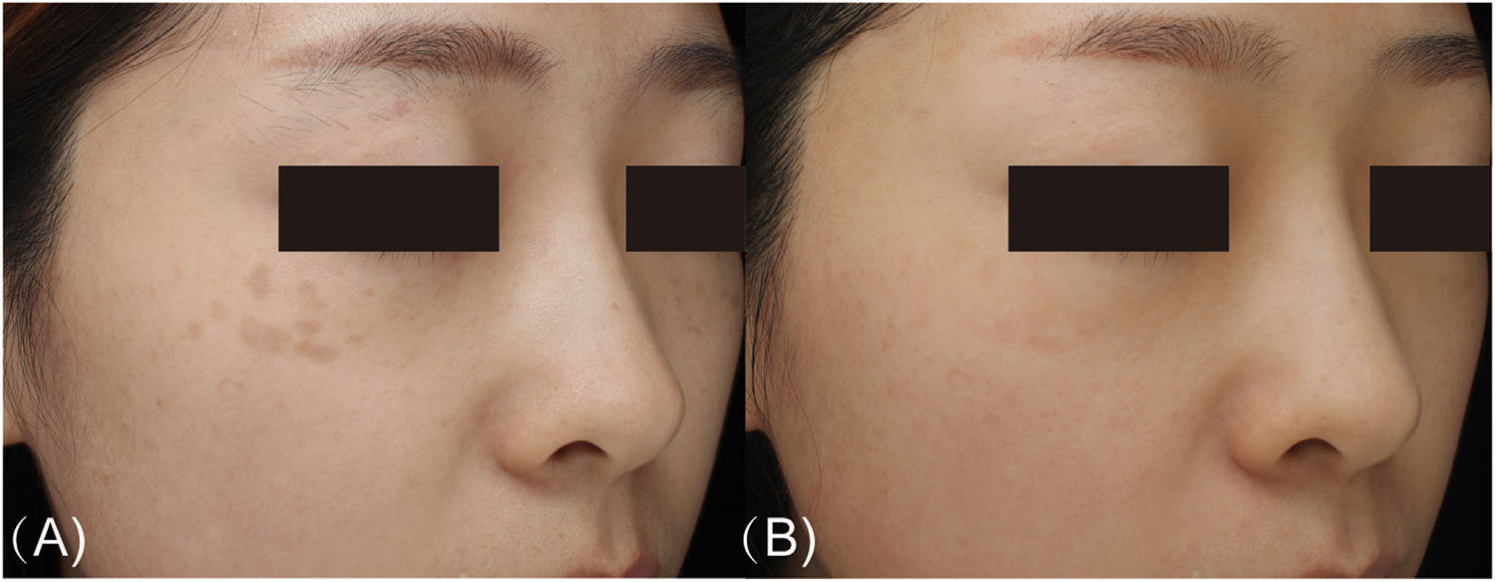
Photographs of a woman in her 20s with only acquired bilateral nevus of Ota‐like macules. (A) Before treatment. (B) Disappearance of all lesions after the fifth treatment session.

Among the 110 patients, the good to excellent clearance rates were 28.1%, 50%, 70%, and 92.3% in the patients treated twice, three times, four times, and five or more times, respectively (Figure 2A); those aged 20 years or younger, 21–29 years, and 30 years or older had good to excellent clearance rates of 86.7%, 64.9%, and 34.3%, respectively (Figure 2B). Photos of two representative cases are shown in Figures 3 and 4. Multiple logistic regression analysis showed that the curative effect was positively correlated with the number of treatment sessions and negatively correlated with the increasing age at first treatment (*p* < 0.05) (Table 3). As the age at the time of first treatment increased, the treatment effect worsened. The treatment effect was better in patients with skin type III than patients with skin type IV (odds ratio: 3.037, 95% confidence interval 1.261–7.313, *p* < 0.05) (Table 3). The curative effect was better in patients with a lesion area of less than or equal to 10 cm^2^ than those with larger lesion areas (*p* < 0.05) and was better in patients without melasma than in those with melasma (odds ratio: 5.940, 95% confidence interval 1.620–21.786, *p* < 0.05) (Table 3). However, the color and number of lesions did not significantly affect the treatment effect (*p* > 0.05) (Table 3). In addition, we further analyzed the relationship between the age of first treatment and the number of treatments to find a potential correlation, and the results showed that the number of treatments was not affected by age (*p* > 0.05).

**TABLE 3:** Multivariate logistic regression analysis of the factors affecting the outcome of Q‐switched Nd:YAG laser treatment for acquired bilateral nevus of Ota‐like macules.

		95%CI			
	OR	Lower limit	Upper limit	*χ* ^2^	*p*
Age (years)					
≤20	5.706	1.076	30.251	4.187	0.041^*^
21–29	3.469	1.171	10.279	5.037	0.025^*^
≥30	1				
Number of treatments					
2	0.005	0.001	0.025	43.758	<0.001^*^
3	0.037	0.009	0.144	22.434	<0.001^*^
4	0.241	0.058	0.996	3.861	0.049^*^
≥5	1				
Fitzpatrick skin type					
III	3.037	1.261	7.313	6.137	0.013^*^
IV	1				
Color					
Yellow–brown	0.645	0.182	2.286	0.461	0.497
Slate–grey	0.480	0.139	1.660	1.345	0.246
Blue–brown	1				
Area (cm^2^)					
≤10	4.784	1.153	13.427	4.784	0.029^*^
11–19	3.315	0.926	8.027	3.315	0.069
≥20	1				
Number of lesions					
1–2	0.687	0.131	3.598	0.198	0.657
3–4	0.546	0.121	2.457	0.621	0.431
5–6	1				
ABNOM with melasma					
No	5.940	1.620	21.786	7.221	0.007^*^
Yes	1				

*Note*: Asterisks indicate statistical significance (*p* < 0.05).

Abbreviations: ABNOM, acquired bilateral nevus of Ota‐like macules; CI, confidence interval; OR, odds ratio.

### 3.3 Adverse reactions

**FIGURE 5:**
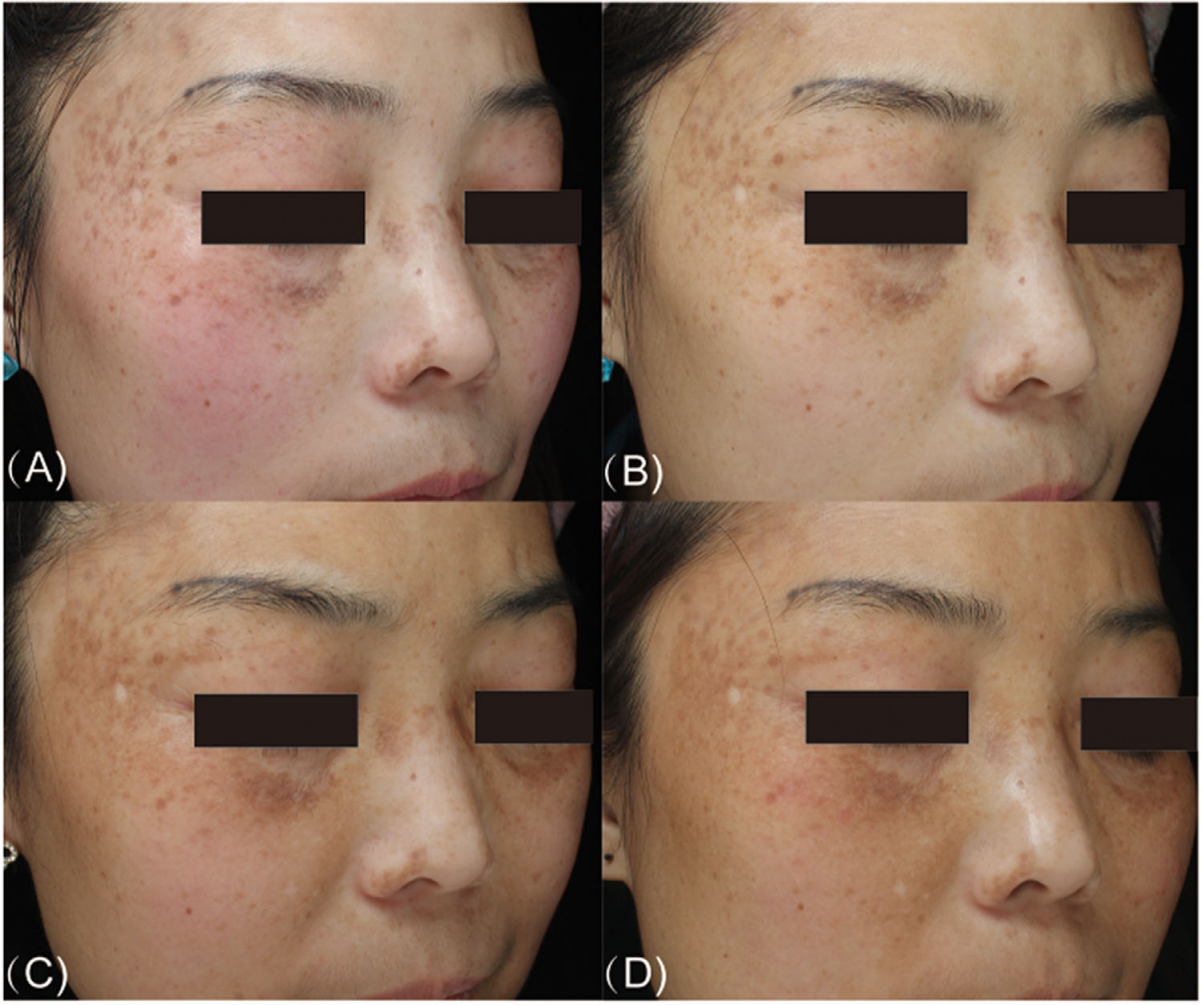
Photographs of a woman in her 30s with acquired bilateral nevus of Ota‐like macules and slight melasma. (A) Before treatment. (B) After the first laser treatment. (C) After the second laser treatment. (D) After the third laser treatment. In the course of three treatments, melasma showed a gradual deterioration trend.

There were no serious adverse reactions during treatment. After treatment, some patients developed temporary mild erythema and edema. No patients developed scarring after treatment. Eleven patients (10.0%) had PIH. PIH had linear relationships with age, skin type, and lesion color (Table 4). The risk of PIH after laser treatment increased with age and was increased in patients with skin type IV and darker lesions (*p* < 0.05). PIH disappeared within 6 months after laser treatment. There were 18 (16.4%) patients with ABNOM who initially had melasma; after laser treatment, 6 (33.3%) had hyperpigmentation, whereas 9 (50%) had aggravation of the original melasma (Figure 5). The continuous correction *χ* ^2^ test showed that patients with melasma were significantly more likely to have PIH than those without melasma (33.3% vs. 5.4%, *p* < 0.05).

**TABLE 4:** Assessment of the linear relationship between each factor and postinflammatory hyperpigmentation (PIH) using the Mantel–Haenszel *χ* ^2^ test.

		PIH				
	Total (*N* = 110)	Yes (*N* = 11)	No (*N* = 99)	*χ* ^2^	*r*	*p*
Age (years)				5.047	0.215	0.025^*^
≤20	15	1	14			
21–29	57	2	55			
≥30	38	8	30			
Fitzpatrick skin type				5.072	0.216	0.024^*^
III	45	1	44			
IV	65	10	55			
Color				8.777	0.284	0.003^*^
Yellow–brown	54	2	52			
Slate–grey	29	2	27			
Blue–brown	27	7	20			
Area (cm^2^)				0.762	0.084	0.383
≤10	36	2	34			
11–19	40	5	35			
≥20	34	4	30			
Number of lesions				1.933	0.133	0.164
1–2	62	4	58			
3–4	36	5	31			
5–6	12	2	10			
Number of treatments				0.701	0.080	0.403
2	32	2	30			
3	32	3	29			
4	20	3	17			
≥5	26	3	23			
ABNOM with melasma				10.104	–	0.01^*^
No	92	5	87			
Yes	18	6	12			

*Note*: Asterisks indicate statistical significance (*p* < 0.05).

Abbreviations: ABNOM, acquired bilateral nevus of Ota‐like macules;PIH,postinflammatory hyperpigmentation; *r*, Pearson correlation coefficient.

## 4 DISCUSSION

**FIGURE 6:**
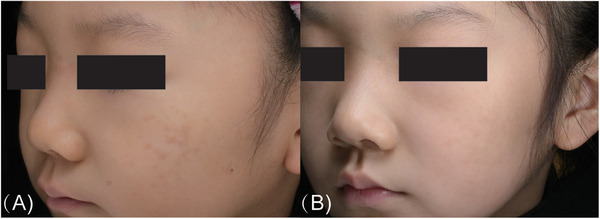
Photographs of a 5‐year‐old girl with acquired bilateral nevus of Ota‐like macules. (A) Before treatment. (B) Good clearance of the lesions after the sixth treatment session.

ABNOM is a common dermal melanocytic hyperpigmentation that was first reported in 1984 and is also called Hori's nevus.^1^ The lesions in ABNOM are round, oval, and polygonal, with clear boundaries. They are mainly distributed in the bilateral zygomatic regions but may also involve the forehead, temporal area, eyelids, and root or alae of the nose^1^, ^2^; the skin above the upper lip is involved in some cases.^16^ Patients with ABNOM have brown, blue–brown, or slate‐gray macules. Our study showed that as the patient age increased, the lesion color gradually deepened and the number of lesion sites increased, which is in agreeance with the findings of previous studies.^17^ Previous studies have shown that ABNOM often occurs after the age of 20 years, but there are also reported cases of ABNOM onset in children aged 9^3^ and 6 years.^16^ One of our patients was only 5‐year old at the time of first presentation at our hospital for treatment (Figure 6).

The etiology of ABNOM is still unclear, but the mechanism is likely to be activation of preexisting dermal melanocytes.^18^, ^19^ The potential activators are sun (ultraviolet) exposure,^2^, ^18^, ^20^ inflammatory reaction,^3^, ^19^ and changes in sex hormone concentrations.^16^, ^18^, ^21^ In our study cohort, there were far more women with ABNOM than men, especially women older than 20 years, which suggests that estrogen plays a role in the development of ABNOM. The association between ABNOM and estrogen was further suggested by the high incidence of melasma induced by laser therapy in our cohort.

Our study evaluated the safety and efficacy of 1064‐nm QSNYL treatment among 110 patients with ABNOM on the basis of the age at first treatment, number of treatments, skin type, lesion color, affected area, number of lesions, and presence of concomitant melasma. The present results showed that the therapeutic effect was related to the number of treatments, age at first treatment, skin type, and lesion area. However, the therapeutic effect was not correlated with lesion color or number of lesion sites. The treatment effect was best in younger patients with a lesion area of less than 10 cm^2^. Previous studies have shown that melanin remains in the exposed area even after laser pulses destroy dermal melanocytes, as the lesion area only lightens after the melanin is engulfed by macrophages and is removed to lymph nodes or other parts.^[^ ^22^ ^]^ As the patient age increased, the lesion area increased and the lesions tended to fuse and darken, which means that the dermal melanin in the lesions increased. We speculate that this situation increases the burden of macrophages to remove melanin after laser treatment, preventing all melanin damaged by laser irradiation from being transported to lymph nodes. Therefore, additional treatment may be required. In addition, our study showed that with increasing age, patients with ABNOM had an increased risk of concomitant melasma and the lesion color tended to darken; both of these factors increase the risk of PIH, which increases the difficulty of treatment and reduces the curative effect. Therefore, to achieve a good therapeutic effect, we suggest that ABNOM should be actively treated in the early stage.

PIH is a frequent problem after laser therapy and is much more problematic in dark‐skinned patients with ABNOM than in those with light skin.^5^, ^9^, ^10^ In our study, patients with skin type inned patients with ABNOM than. PIH increases the patient's psychological burden, reduces their satisfaction with treatment, and affects their confidence in continuing treatment. The pathogenesis of PIH includes the presence of latent dermal melanocytosis, which is triggered by extrinsic stimulants such as hormones, sunlight, and trauma. Laser therapy is not recommended in patients with a recent history of sun exposure or administration of photosensitive drugs. Additionally, as the melanocytes in ABNOM are often distributed in the perivascular area, direct injury to these melanocytes may cause indirect vascular injury and induce an inflammatory response.^23^, ^24^ Thus, the onset of PIH is strongly correlated with the severity of prior inflammation. A higher energy density is more likely to cause an excessive inflammatory reaction. Kunachak et al. used higher fluence of 8–10 J/cm^2^ for treatment (*n* = 70, 1064 nm, 2–4 mm, 10 Hz), and hyperpigmentation after treatment occurred in 50% of patients.^[^ ^9^ ^]^ Suh et al. used QSNYL treatment, with an average fluence of 7.8 J/cm2 (*n* = 10, 1064 nm, 3 mm, 10 Hz), and obtained similar results. Almost all patients had transient PIH.^10^ In contrast, Cho et al. described the effect of 1064‐nm QSNYL with low fluence in the treatment of ABNOM (*n* = 15, 2.2–6.0 J/cm^2^). After 10–15 times of treatment, there is no case of hypopigmentation or hyperpigmentation, and the overall satisfaction is high.^11^ In our study, we used a lower energy density fluence to reduce the local inflammatory response of patients, especially for patients with older age, darker skin and lesion color, and concomitant melasma. Before laser treatment, all patients were informed that they may develop PIH and were advised to strictly avoid sunlight to minimize the risk of PIH. The treatment interval was prolonged in patients who developed PIH. Patients were also advised to moisturize and strengthen sunscreen measures. The incidence of PIH after laser treatment in our study was 10%, which is less than the incidence of 50% and 100% in previously reported.^9^, ^10^ This is inseparable from strengthening the evaluation of patients’ condition before laser treatment and selecting appropriate energy density. In our study cohort, PIH subsided spontaneously within 3–6 months in all affected patients. Oral vitamin C and topical hydroquinone cream were applied to accelerate the regression of pigmentation. The laser treatment was continued after the PIH disappeared.

Our study found that the melasma worsened after treatment in 9 (50%) of the 18 patients with ABNOM concomitant melasma, whereas 2 (2.2%) patients developed new melasma after treatment. The pathogenesis of melasma involves many factors, such as ultraviolet radiation, estrogen, cosmetics, emotional factors, and oral contraceptives. Phototoxic stimulation may also be responsible for the worsening of melasma. Wang et al.^12^ speculated that melasma and ABNOM have the same origin. Hence, laser irradiation may push the development of the disease in another direction and induce melasma. ABNOM treatment should be initiated as early as possible and before the age of 35 years, when the risk of inducing melasma is very low. In our clinical practice, the laser energy is reduced to 2.5–4.0 J/cm^2^ for patients with melasma to reduce the risk of aggravation. When there is aggravation or new onset of melasma, we prolong the treatment interval. Oral tranexamic acid is usually recommended, and laser irradiation is not performed in areas with melasma.

Our study has some limitations. It was a retrospective study with the limitations of a small sample size and lack of objective evaluation. Future large‐scale prospective studies are warranted to evaluate the effectiveness and safety of QSNYL therapy for ABNOM.

## 5 CONCLUSION

ABNOM occurs more frequently in women than in men and is effectively and safely treated with 1064‐nm QSNYL therapy. Earlier initiation of laser treatment achieves a better treatment effect, and the curative effect is better in patients with an affected area of less than 10 cm^2^. The risk of PIH after laser treatment is highest in patients with older age, darker lesion color, and darker skin color. Patients with melasma have a poor curative effect and are prone to PIH and melasma aggravation. For such patients, we recommend using a lower energy for laser treatment.

## CONFLICT OF INTEREST STATEMENT

The authors have no relevant financial or nonfinancial interests to disclose.

## ETHICS STATEMENT AND CONSENT TO PARTICIPATE

This study was approved by the Ethics Committee of Provincial Hospital Affiliated to Shandong First Medical University according to the Declaration of Helsinki principles (SWYX: no. 2022‐171). All patients or parents of the child participants provided written informed consent.

## PHOTO CONSENT STATEMENT

I confirm that written consent for permission to publish was obtained from parent(s)/guardian(s) and/or participants to publish any recognizable images of people.

## Data Availability

The datasets used and/or analyzed in the current study are available from the corresponding authors upon reasonable request.
